# Critical Appraisal of “A Comprehensive Analysis of Moist Versus Non‐Moist Dressings for Split‐Thickness Skin Graft Donor Sites”

**DOI:** 10.1002/hsr2.70987

**Published:** 2025-07-02

**Authors:** Ayesha Khaliq, Ashfaq Ahmad, Javed Iqbal

**Affiliations:** ^1^ Karachi Medical and Dental College Karachi Pakistan; ^2^ Gomal Medical College D.I.Khan Dera Ismail Khan Pakistan; ^3^ Nursing Department, Hamad Medical Corporation Doha Qatar

To the Editor,

I am writing to respond to the recently published study, “*A Comprehensive Analysis of Moist Versus Non‐Moist Dressings for Split‐Thickness Skin Graft Donor Sites*,” randomized patients to determine the best donor site care for STSG. This reveals that moist dressings promote faster healing with fewer complications than non‐moist dressings applied to skin defects. Its comprehensive literature review and appropriate inclusion criteria bolster its conclusions. However, I observed a few methodological and reporting shortcomings in this study, which, if rectified, could improve the study's scientific rigor and clinical importance. I share five specific recommendations below, followed by supporting academic literature.

First, the study considers dressing change frequency as an outcome, but it is unclear whether consistent, standardized protocols were used across centers. Whether a fixed schedule, clinical need, or provider choice drove dressing changes is unknown. Differences in the frequency of dressing changes could also bias results because some dressings need to change more frequently regardless of the healing efficacy. Moreover, the study does not include a cost analysis of dressing changes, which is important for determining clinical feasibility in resource‐limited settings. Reporting how dressings were monitored and replaced, including costs, would increase the generalizability of the study [1]. For instance, recording average changes per patient and associated labor costs could guide providers (Adding such data could help hospitals plan budgets for wound care supplies).

Secondly, the study does not address wound healing variables such as smoking, diabetes, and nutritional status. These comorbidities significantly impact re‐epithelialization and infection risk, which may confound the comparison between moist and non‐moist dressings [2]. For example, diabetes reduces the rate of healing due to impaired angiogenesis, while smoking reduces tissue oxygenation, both of which may obscure dressing‐specific effects [2]. This limitation could have been mitigated by using a statistical model like multivariate regression to adjust for these factors and improve the study's validity. Stratifying patients by comorbidity status could further elucidate whether dressing efficacy varies across high‐risk groups and ensure that results are generalizable to different clinical populations (For instance, analyzing diabetic patients separately could show if moist dressings remain effective despite delayed healing).

Third, the study does not explore subgroup analyses according to anatomical location or wound size, which may affect healing outcomes. Scalp donor sites differ from thigh donor sites because of their vascularity and skin thickness. Moreover, wound size can affect dressing performance, with more extensive wounds potentially having different exudate levels and tension than smaller ones [3]. If the data is pooled, subgroup analysis could identify whether moist dressings are superior to non‐moist dressings across anatomical sites or wound sizes. This data would instruct clinicians to select dressing choices specific to the donor site, optimizing outcomes (Such analyses could reveal, for example, if scalp sites heal faster with moist dressings due to better blood supply).

Fourth, there is a significant gap in data regarding long‐term follow‐up on donor site outcomes like hypertrophic scarring and aesthetic quality. The study evaluated only initial re‐epithelialization; however, functional and cosmetic issues are often experienced by donor sites after healing. Scar quality is a heterogeneous entity where dark skin type, female gender, and lower leg sites were found to correlate with poor outcomes like scar color mismatch and elevated scars [4]. Quantifying these effects could be achieved through follow‐up assessments at 1, 3, and 6 months using tools such as the Patient and Observer Scar Assessment Scale (POSAS). This would ensure a broader assessment of how dressings affect healing, extending beyond short‐term wound healing to patient‐centered concerns about long‐term skin appearance (and function).

Lastly, the study does not consider patient preferences and compliance, which are important in postoperative care. Patients value outcomes such as pain relief, comfort, and ease of dressing use, but these were not evaluated. Implementation of a validated patient‐reported outcomes tool like a Likert‐based satisfaction scale would allow these perspectives to be captured [5]. For instance, moist dressings may be less painful during changes but less comfortable if bulky or prone to leakage. Balancing clinical efficacy with usability would inform dressing selection, contributing to understanding patient experiences. Future studies should include such metrics to ensure that dressings meet the needs of patients, increasing adherence to treatment and improving satisfaction (e.g., a scale could measure pain on a 1–5 range to quantify patient comfort).

To conclude, although the study shows that moist dressings are superior to non‐moist dressings for STSG donor site healing, its methodological limitations could be improved to strengthen its impact. Resolving the deficiencies listed, standardizing dressing protocols, controlling for comorbidities, analyzing subgroups, assessing long‐term outcomes, and prioritizing patient perspectives would bolster its conclusions. My appeal is for further research to understand dressing strategies better, ensure effective healing, and address patient needs. This could confirm moist dressings as the standard of care while ensuring that all clinical decisions consider patient comfort and satisfaction.

## Author Contributions

**Ayesha Khaliq:** methodology, validation, visualization, writing – original draft, data curation, and formal analysis. **Ashfaq Ahmad:** writing – original draft, writing – review and editing, methodology, software, formal analysis, and conceptualization. **Javed Iqbal:** conceptualization, investigation, funding acquisition, visualization, project administration, resources, supervision, data curation, and software.

## Ethics Statement

The authors have nothing to report.

## Conflicts of Interest

The authors declare no conflicts of interest.

## Data Availability

This letter is based on publicly available data from the cited publication; no new data were created or analyzed.
